# Spontaneous subdural hematoma in a patient receiving dual antiplatelet therapy following percutaneous coronary intervention: A case report

**DOI:** 10.1016/j.amsu.2021.102941

**Published:** 2021-10-12

**Authors:** Youssra Bouhaddoune, Karima Benbouchta, Noha El Ouafi, Zakaria Bazid

**Affiliations:** a Department of Cardiology, Mohammed VI University Hospital, Faculty of Medicine and Pharmacy of Oujda, Mohammed First University, Oujda, Morocco; b Epidemiological Laboratory of Clinical Research and Public Health, Faculty of Medicine and Pharmacy of Oujda, Mohammed First University, Oujda, Morocco

**Keywords:** Case report, Antiplatelet therapy, Clopidogrel, Intracranial hemorrhage, Subdural hematoma, Acute coronary syndrome

## Abstract

**Introduction:**

and importance: Dual antiplatelet therapy with clopidogrel and aspirin is routinely prescribed after coronary artery stenting, plays a critical role in secondary prevention among patients with acute coronary syndrome and has decreased the rates of re-infarction and stent thrombosis after percutaneous coronary intervention**,** but they are prone to internal bleeding. Intracranial hemorrhage is the most serious bleeding complication in a patient put on antiplatelet therapy following PCI. Acute spontaneous subdural hematoma (ASSDH) without trauma is a rare event, which needs to be promptly recognized and managed.

**Case presentation:**

In this mini-review, we report a case of a 71-year-old man who represented spontaneous acute subdural hematoma receiving dual antiplatelet (aspirin and clopidogrel) following percutaneous coronary intervention for acute coronary syndrome. Rapid discontinuation of all of the antiplatelet drugs and hematoma evacuation were performed with good postoperative evolution.

**Clinical discussion:**

Management of hemorrhagic patients under antithrombotic therapy is very difficult. Resuming the treatment could lead to recurrence bleeding, on the other hand, suspension or stopping of treatment could expand the thrombotic risk. ASSDH after PCI is true diagnostic then therapeutic emergency, especially in patients with rapid neurological degradation. Treatment may be managed by nonoperative conservative approach in selected cases.

**Conclusion:**

Spontaneous subdural hematoma is a rare, serious entity, although it can engage the functional and vital prognosis of the patient, hence the interest of diagnosis and prompt treatment to improve the prognosis.

## Highlights

• Acute spontaneous subdural hematoma (ASSDH) is rare, and often-fatal disorder.
• Intracranial hemorrhage it is the most serious bleeding complication in a patient put on antiplatelet therapy following PCI.
• Opportune management and a correct strategy in secondary prevention of bleeding events are crucial factors to decrease morbidity and mortality in these patients and to improve the prognosis.
• Surgical interventions are often essential for large or rapidly expanding hematomas, with significant mass effects.

## 1 Introduction

Percutaneous coronary intervention (PCI) and dual antiplatelet therapy are common management for patients with acute coronary syndrome. Nonetheless, those treatments can provoke serious complications such as bleeding or hematomas at various anatomic sites and intracranial hemorrhage is the most feared complication of antithrombotic therapy and it can increase the risk of in-hospital death by 60% [1]. Acute subdural hematomas are rarely reported in the literature, are generally associated with traumatic brain injury. Spontaneous acute subdural hematoma without trauma is an uncommon event, but it is a serious condition. In this mini-review, we report a case of a spontaneous acute subdural hematoma in an elderly man receiving dual antiplatelet with clopidogrel and aspirin following percutaneous coronary intervention for acute coronary syndrome, emergency surgery was successfully performed and the patient recovered well. Early diagnosis and prompt treatment of this complication are the keys to improving the prognosis.

Our case report was written according to SCARE guidelines [2].

## 2 Case presentation

We report the case of a 71-year-old man, with a past medical history of diabetes mellitus, without history of head trauma nor other medical, surgical, family, psychosocial, and pharmacologic histories or vascular anomaly. He was admitted to the emergency room with sudden onset retrosternal pain with radiation to back appeared for 14 hours. At presentation, he was conscious (Glasgow coma scale 15 points), oriented and cooperative, her pulse was 110 beats per min, and her blood pressure was 120/70 mmHg. Cardiovascular examination was unremarkable.

**Fig. 1:**
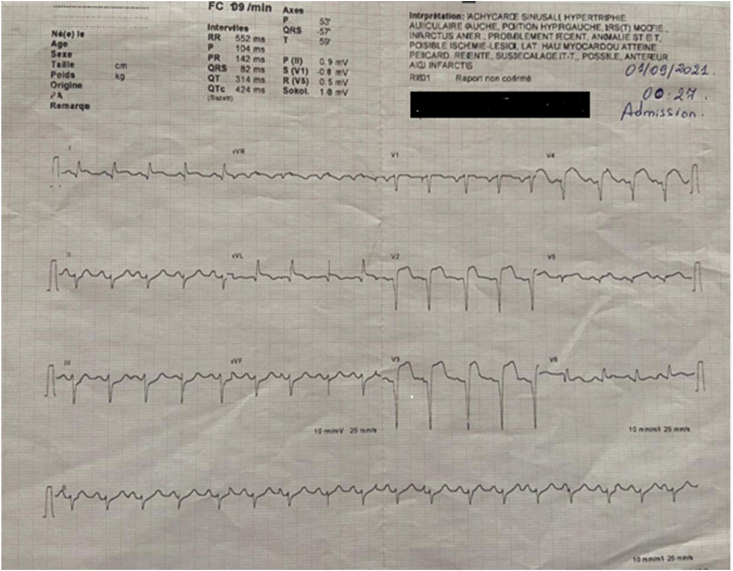
12-Lead ecg revealed extensive anterior St-elevation myocardial infarction (In leads: I, Avl and V1–V6).

**Fig. 2:**
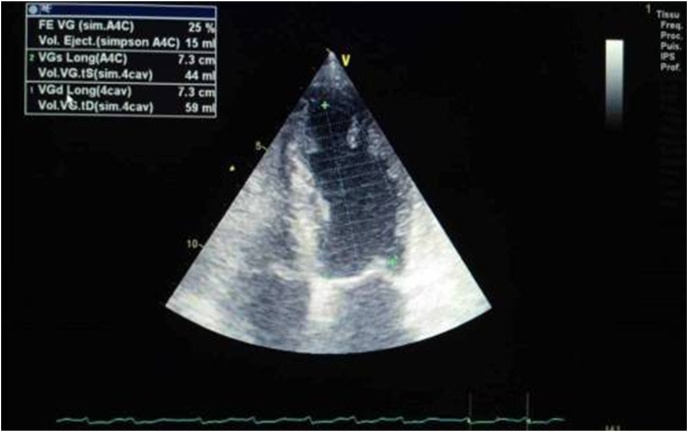
Transthoracic echocardiography showing severe systolic dysfunction at 25%.

**Fig. 3:**
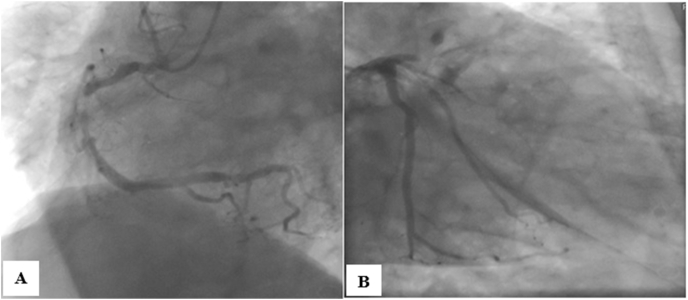
A) Coronary angiography showing severe stenosis of the right coronary artery **B**) Occlusion in the proximal segment of the left anterior descending artery, severe stenosis of the main of the marginal artery.

**Fig. 4:**
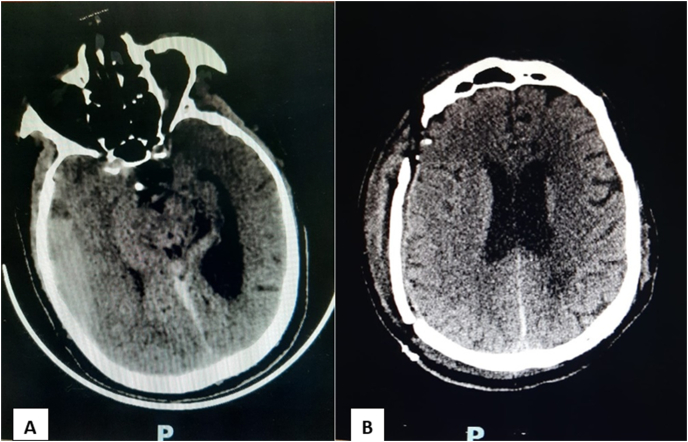
A) Preoperative brain computed tomography scan revealing a right temporoparietal acute subdural hematoma with mild mass effect on the ipsilateral ventricle. **B)** Postoperative brain computed tomography scan revealing complete resolution of subdural hematoma.

Admission ECG showed acute extensive anterior myocardial infarction (Fig. 1). Initial laboratory data revealed an elevated white blood cells count of 15,870/μL (4000–1000/μL), C-reactive protein at 2.23 mg/L, hemoglobin at 16.5 g/dl, and a normal platelet count at 205.000/μL (150,000 à 400,000/μL), partial thromboplastin time of 25 seconds (24–41), prothrombin time of 10 seconds (9–12), and an international normalized ratio (INR) of 1, renal and liver function tests were within normal. Transthoracic echocardiography revealed akinesia in the anterior wall and the apex with severe left ventricular systolic dysfunction with ejection fraction of 25% (Fig. 2). Diagnosis of acute ST-elevation elevation myocardial infarction was made, and the patient received pharmacological measures (aspirin 300mg, clopidogrel 300mg and low-molecular-weight heparins). Coronary angiography revealed atherosclerotic coronary disease with occlusion of the left anterior descending coronary artery, severe stenosis of the main in the marginal artery, and severe stenosis in the right coronary artery (Fig. 3 A, B). The patient underwent primary percutaneous coronary intervention (PCI) with placement of five stents in total, without peri-procedural complications. Dual antiplatelet therapy (a aspirin at a dose of 75mg once daily and clopidogrel at a dose of 75 mg once daily) continued after percutaneous coronary intervention. After the procedure, his vital signs and neurological status remained stable. Twenty-four hours later of hospitalization, the patient became confused with GCS of E4V4M6. At neurological examination he was disoriented, confused, there were no focal neurological deficits. Brain computed tomography (CT) scan revealed a right temporoparietal acute subdural hematoma measuring 17 mm with mild mass effect on the ipsilateral ventricle (Fig. 4 A), there was no evidence of fracture or external contusion, rest of the brain parenchyma was normal. Laboratory investigations including hemogram, INR, activated partial thromboplastin time (APTT) were normal. The patient was diagnosed with acute subdural hematoma.

After a discussion among cardiologists, neurosurgeon, and anesthesiologists, the decision was to stop all of the antiplatelet drugs. The patient was directly taken to the operating room, where a surgical evacuation of the hematoma was performed successfully by an experienced professor of neurosurgery with the aid of an junior trainee with 3 years of surgical specialty training (using general anesthesia). The intervention adherence and tolerability were well during the whole course of his treatment. After evacuation of the subdural hematoma, the patient was admitted to intensive care unit. Three day later, control brain computed tomography scan revealing complete resolution of subdural hematoma (Fig. 4 B). After 7 days, in the absence of any clinical, biological or radiological degradation, cardiology and neurosurgeon decided to start an aspirin mono-therapy antiplatelet agent-based, since the risk of stent thrombosis was high and he could be discharged from our hospital in good health without any neurological deficit 9 days after admission. One month after the discharge, the patient had no other bleeding episodes under aspirin treatment or thrombotic events. Control brain computed tomography scan showed that the subdural hematoma had completely resolved.

## 3 Discussion

Acute subdural hematoma is generally associated with traumatic brain injury, acute spontaneous subdural hematoma (ASSDH) without trauma is rare, serious entity. Dual antiplatelet therapy with clopidogrel and aspirin is routinely prescribed after coronary artery stenting, plays a critical role in secondary prevention among patients with acute coronary syndrome and has decreased the rates of re-infarction and stent thrombosis after percutaneous coronary intervention. The incidence of bleeding complication are respecting 4.1–9.0% [3]. Factors that might contribute to its development include hypertension [4] aneurysms [5] arterio-venous malformations [6], and cocaine usage [7], pharmacologic therapy linked with the development of spontaneous acute spontaneous subdural hematoma comprise aspirin, heparin, and warfarin [8]. Our patient had a acute subdural hematoma after PCI under dual antiplatelet therapy, however, he did not show any signs of coagulopathy, thrombocytopenia or head trauma. Antiplatelet agents are frequently used and their hemorrhagic complications occur usually at skin or gastrointestinal sites. Serious bleeding event can occur with antiplatelet therapy and intracranial hemorrhage is the most feared complication of antithrombotic therapy and may lead to severe morbidity and mortality unless diagnosed and treated early [9,10]. Therefore, early diagnosis and treatment of subdural hematoma is very important because the mortality rate is estimated at 60%–76.5% of cases [4]. Here, we describe a case of spontaneous acute subdural hematoma in a patient with ischemic heart disease receiving dual antiplatelet therapy (with Aspirin and clopidogrel) following percutaneous coronary intervention. He recuperated with no residual neurological deficits. Rapid discontinuation of all of the antiplatelet drugs and hematoma evacuation were performed with a favorable evolution. Early diagnosis and prompt treatment of this complication resulted in a good result in this patient. Clinical presentation of SDH may be variable, and it depends on the hematoma volume, usually manifests as vomiting, acute headache, neurological deficits or sudden unexplained coma [8]. Surgical evaluation should be urgent and a brain CT scan be performed as quickly as possible. Connolly et al. [11] have reported on a meta-analysis of randomized clinical trials the danger of subdural hematoma associated with aspirin therapy. In four published clinical trials with 6565 participants with 8 total subdural hematomas. Unpublished data of 5 clinical trials with 90,689 participants described eighteen subdural hematomas. The incidence of subdural hematomas was 0.02/1000 patient-years for primary prevention trials of middle-aged health professionals to 1 to 2/1000 patient-years on aspirin with odds ratio of 1.6. Wong et al. [12] remark that location of lobar hematoma was higher in the aspirin group (32.8%) than in the control group (10.3%).

Bakheet et al. [13] have described on a meta-analysis of 11 randomized clinical trials analyzing the risk the danger of subdural hematoma in a patient receiving dual antiplatelet therapy (clopidogrel plus aspirin). Though 8 trials did not show any case of spontaneous subdural hematoma, three trials with 23,136 participants reported 39 subdural hematomas during a mean follow-up 2.1 years per patient. They concluded that the absolute rate of subdural hematoma in patients on dual antiplatelet therapy is weak averaging 1.1/1000 patient-years. there was no elevated risk of spontaneous acute subdural hematoma in-patient on dual antiplatelet therapy. Nonetheless, there was a higher risk of major bleeding with clopidogrel plus aspirin compared with aspirin plus placebo or aspirin alone.

Management of hemorrhagic patients under antithrombotic therapy is very difficult. Resuming the treatment could lead to recurrence bleeding, on the other hand, suspension or stopping of treatment could expand the thrombotic risk. Early diagnosis and surgical intervention are often essential for hematomas with significant mass effects. Treatment may be managed by nonoperative conservative approach in selected cases. Strategies recommended [14] in reducing the risk and manage bleeding complications comprehend utilize of low dose aspirin, limiting the use of theinopyridine therapy following PCI to 2 weeks for angioplasty, for 4 weeks in cases of a bare stent, and for 12 months in cases of a drug –eluting stent, restart DAPT with aspirin plus clopidogrel, one month after intracranial hemorrhage in acute coronary syndrome patients with drug-eluting stent implantation shorter than 3 months ago and continue DAPT until the minimal advised duration [15,16]. In our case, rapid discontinuation of all of the antiplatelet drugs and surgical hematoma evacuation were performed with a favorable evolution.

The patient was seen in the consultation of our university hospital one month after the discharge, the patient had no other bleeding episodes under aspirin treatment or thrombotic events. Control brain computed tomography scan showed that the subdural hematoma had completely resolved.

Our patient was satisfied with the quality of our management.

The limitations of this are that even though we throw away the possibility of a minor trauma and meticulously instruct the patient, there is not enough to eliminate the possibility of chronic subdural hematoma as a result of an accidental minor injury to the head.

## 4 Conclusion

Acute subdural hematomas in acute coronary syndromes are scarce but critical conditions after percutaneous coronary intervention (PCI). The use of this antiplatelet therapy requires the cardiologist be vigilant of this possible side effect. Opportune management and a correct strategy in secondary prevention of bleeding events are crucial factors to decrease morbidity and mortality in these patients and to improve the prognosis.

## Ethical approval

The ethical committee approval was not required give the article type (case report). but we notice that the written consent to publish the clinical data of the patients was given and is available to check by the handling editor if needed.

## Sources of funding

This research did not receive any specific grant from funding agencies in the public, commercial, or not-for-profit sectors.

## Author contribution

Youssra Bouhadoune: Study concept, Data collection, Data analysis, Writing the paper, Karima Benbouchta: Data analysis, El Ouafi Nouha: Conception, methodology, supervision, Bazid Zakaria: Conception, methodology, supervision.

## Research registration

This is a medical case report not an original research project. This registration is not required.

## Guarantor

Youssra Bouhadoune.

## Consent

Written informed consent was obtained from the patient for publication of this case report and accompanying images. A copy of the written consent is available for review by the Editor-in-Chief of this journal on request.

## Provenance and peer review

Not commissioned, externally peer-reviewed.

## Declaration of competing interest

There are no conflicts.
